# Active propagation of dendritic electrical signals in *C*. *elegans*

**DOI:** 10.1038/s41598-019-40158-9

**Published:** 2019-03-05

**Authors:** Tomomi Shindou, Mayumi Ochi-Shindou, Takashi Murayama, Ei-ichiro Saita, Yuto Momohara, Jeffery R. Wickens, Ichiro N. Maruyama

**Affiliations:** 1 Neurobiology Research Unit, Okinawa Institute of Science and Technology Graduate University, Okinawa, 904-0495 Japan; 2 Information Processing Biology Unit, Okinawa Institute of Science and Technology Graduate University, Okinawa, 904-0495 Japan

## Abstract

Active propagation of electrical signals in *C*. *elegans* neurons requires ion channels capable of regenerating membrane potentials. Here we report regenerative depolarization of a major gustatory sensory neuron, ASEL. Whole-cell patch-clamp recordings *in vivo* showed supralinear depolarization of ASEL upon current injection. Furthermore, stimulation of animal’s nose with NaCl evoked all-or-none membrane depolarization in ASEL. Mutant analysis showed that EGL-19, the α1 subunit of L-type voltage-gated Ca^2+^ channels, is essential for regenerative depolarization of ASEL. ASEL-specific knock-down of EGL-19 by RNAi demonstrated that EGL-19 functions in *C*. *elegans* chemotaxis along an NaCl gradient. These results demonstrate that a natural substance induces regenerative all-or-none electrical signals in dendrites, and that these signals are essential for activation of sensory neurons for chemotaxis. As in other vertebrate and invertebrate nervous systems, active information processing in dendrites occurs in *C*. *elegans*, and is necessary for adaptive behavior.

## Introduction

Sensory information is commonly coded by the frequency and timing of action potentials^1,2^, whereas many sensory neurons, including crustacean stretch receptors, insect and vertebrate retinal photoreceptors and mammalian hair cells, use graded potentials^3,4^. High-impedance neurons with short processes such as *C*. *elegans* neurons are also thought to transmit electrical signals by passive propagation. Indeed, a lack of channels necessary for Na^+^-dependent action potentials^5,6^ has previously led to an emphasis on passive current spread as a mechanism for electrical signal propagation in *C*. *elegans* neurons^7–12^. Graded electrical potentials and graded synaptic transmission in motor neurons are also observed in *C*. *elegans*^13^ and the parasitic nematode, *Ascaris suum*^14,15^. Upon current injection, however, non-linear depolarization is observed in a gustatory sensory neuron, ASER, in *C*. *elegans*^16^. Furthermore, when depolarizing current is artificially injected, regenerative potentials with all-or-none properties have been reported in *C*. *elegans* RMD neurons^17^.

Calcium-dependent action potentials have been recorded in *C*. *elegans* pharyngeal muscles^18,19^ and body-wall muscles^20,21^. Voltage-gated Ca^2+^ channels (VGCCs) can also generate spikes in dendrites of rodent neocortical pyramidal neurons^22,23^. VGCCs of vertebrates have been classified into three subtypes, based on structural, functional, kinetic, and pharmacological differences^24^. In particular, the α1 subunits of Cav1, Cav2, and Cav3 calcium channels are key determinants of the three calcium channel subtypes L-type, R,N,P/Q-type and T-type, respectively. In *C*. *elegans*, three genes, *egl-19*, *unc-2* and *cca-1*, encode pore-forming L-type, R,N,P/Q-type and T-type α subunits of VGCCs, respectively^19,25,26^. In *C*. *elegans* body-wall muscles, the L-type VGCC in particular, and extracellular Ca^2+^ are essential to generate all-or-none action potentials^20,21^.

The bilaterally symmetric pair of bipolar head neurons, ASEL and ASER, are major gustatory chemosensory neurons in *C*. *elegans*, and terminate with ciliated endings in the animal’s nose, which is exposed to the environment^27,28^. Various chemicals, including Na^+^, K^+^, Cl^−^, Mg^2+^, Li^+^, alkaline pH, cAMP, and biotin, are sensed by the ASEL and ASER neuron pair^29–32^. Increased or decreased NaCl concentrations are sensed, respectively, by ASEL and ASER^31,32^. An increase in environmental NaCl concentration is detected partly by the receptor guanylyl cyclase GCY-14 in ASEL, which produces cGMP^30^. Upon stimulation of ASEL with NaCl, Ca^2+^ transients observed in the cell body are defective in *tax-2* or *tax-4* animals, suggesting that ASEL is activated by Ca^2+^ influx through TAX-2/TAX-4 cGMP-gated cation channels^13,30,33,34^. In previous electrophysiological studies, ASER was reported to be nearly isopotential and unable to generate classical Na^+^ action potentials^16^. However, it is still not known whether ASEL is also isopotential in response to environmental increases in NaCl concentration.

Here, we show that an environmental increase in NaCl concentration induces Ca^2+^ influx through TAX-2/TAX-4 cGMP-gated channels into ASEL sensory endings. The resulting membrane depolarization activates EGL-19 VGCC for regeneration of all-or-none electrical signals in ASEL dendrites. It demonstrates that the small, simple nervous system of *C*. *elegans* uses mechanisms of information processing similar to those of other invertebrate and vertebrate brains, in which both active and passive electrical signal transmission are used^35,36^.

## Results

### Supralinear depolarization of ASE sensory neurons upon current injection

**Figure 1:**
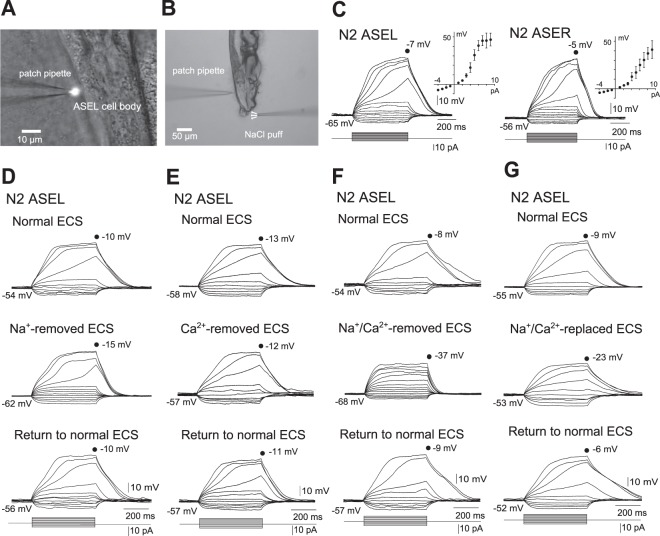
Whole-cell, current-clamp recordings *in vivo* of ASE neurons in wild-type N2 animals. (**A**) Image of a patch-clamp recording. *gcy-7*p*::GFP*-transfected N2 specifically producing GFP in ASEL was glued to a cover glass, and was transferred to a recording chamber. Under a microscope, a small piece of cuticle and body wall was dissected to exteriorize ASEL cell bodies. (**B**) Image of an animal under stimulation with NaCl-containing buffer. Animals were treated with the same procedures as in (**A**), and their nose tips were stimulated with NaCl solutions released from a puffing capillary. (**C**) Membrane voltage changes in response to current steps and averaged *I-V* curves in wild-type ASEL (left, *n* = 22) and ASER (right, *n* = 7). Error bars, s.e.m. (**D**–**G**) Membrane voltage changes in response to current steps in wild-type ASEL. Current-clamp recordings of an animal were carried out three times consecutively, first and third in normal ECS (top and bottom), and the second in ECS, from which Na^+^ was removed (Na^+^-removed ECS) (**D**), Ca^2+^ of which was removed (Ca^2+^-removed ECS) (**E**), Na^+^ and Ca^2+^ of which were removed (Na^+^/Ca^2+^-removed ECS. In this experiment, current was injected until the membrane potential exceeded −40 mV) (**F**), or Na^+^ and Ca^2+^ of which were replaced with NMDG^+^ and Mg^2+^ (Na^+^/Ca^2+^-replaced ECS) (**G**). These are representatives of at least three independent recordings.

We first tested whether regenerative membrane depolarization occurs in ASE neurons in response to current steps, using *in vivo* whole-cell patch-clamp recordings (Fig. 1A) of a slit-worm preparation^16^. ASEL and ASER had resting membrane potentials of −61.7 ± 1.1 mV (*n* = 22) and −56.6 ± 1.0 mV (*n* = 7), and input resistances 2.2 ± 0.2 GΩ (*n* = 22) and 1.6 ± 0.2 GΩ (*n* = 7), respectively (Fig. 1C). These resting potentials were comparable to those observed previously in ASER^16^ and RMD^17^. In response to depolarizing current steps, membrane potentials of both ASEL and ASER neurons depolarized linearly at lower current intensities. Importantly, the voltage trajectories of both cell types became supralinear at higher currents, and reached a plateau during the depolarization phase as shown in steady-state current-voltage (*I-V*) plots (Fig. 1C). Such non-linear depolarization has also previously observed in ASER^16^, but has not previously been reported in ASEL.

In RMD neurons, the amplitude of nonlinear depolarization was reduced when the chamber solution was replaced with Na^+^-free, or Na^+^- and Ca^2+^-free extracellular saline (ECS)^17^. To identify ion species required for the supralinear depolarization of ASEL, we removed Na^+^ and/or Ca^2+^ from the ECS (Fig. 1D–G). When either Na^+^ or Ca^2+^ alone was removed from ECS, supralinear depolarization was still observed in response to steps of depolarizing current (Fig. 1D,E). When Na^+^ or Ca^2+^ was replaced with NMDG^+^ or Mg^2+^, respectively, in ECS, supralinear depolarization still remained (Supplementary Fig. S1). When both Na^+^ and Ca^2+^ were removed from ECS, in contrast, supralinear depolarization disappeared (Fig. 1F). In addition, when both Na^+^ and Ca^2+^ were replaced with NMDG^+^ and Mg^2+^ in ECS, supralinearity was markedly reduced (Fig. 1G). Complete recovery from all these changes was observed following a return to normal ECS. These results suggest that either Na^+^ or Ca^2+^ is required for supralinear depolarization of ASEL upon current injection, as previously observed in RMD neurons^17^.

### All-or-none membrane depolarization evoked in ASEL by NaCl

**Figure 2:**
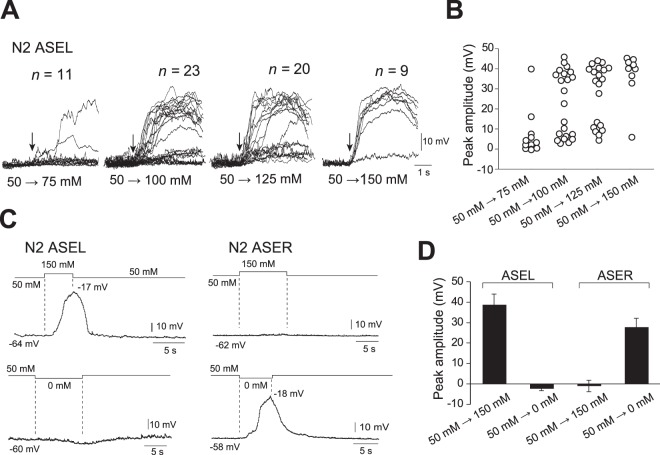
Membrane depolarization evoked by application of various concentrations of NaCl to the nose tips of animals. (**A**) Membrane voltage traces of ASEL in response to 75 mM, 100 mM, 125 mM, or 150 mM NaCl. An experimental setting is shown in Fig. 1B. Vertical arrows indicate onset times of NaCl-evoked depolarization. Voltage traces from different animals were superimposed by synchronizing onset times at the same position. (**B**) Peak amplitudes, which are shown by circles, of membrane voltage traces shown in (**A**). (**C**) Membrane voltage traces of ASEL and ASER in response to application of 150 mM NaCl or NaCl-free buffer, respectively. Note that ASEL and ASER responded to increases and decreases of NaCl concentration, respectively. (**D**) Mean amplitudes of membrane voltage peaks in response to 150 mM NaCl or NaCl-free buffer (ASEL: *n* = 7 up-steps, *n* = 3 down-steps; ASER: *n* = 4 up-steps, *n = *6 down-steps). Error bars, s.e.m.

Whole-cell recordings from wild-type N2 animals (Fig. 1B) showed that ASEL depolarized in response to a puff of various concentrations of NaCl to animal’s nose in ECS containing 50 mM NaCl (Fig. 2A). This depolarization continued during the application of NaCl, and after the cessation of NaCl application, the membrane potential repolarized to resting levels (Fig. 2C, left). Conversely, a puff of NaCl-free buffer induced depolarization of ASER membrane potentials (Fig. 2C, right). The ECS was perfused from the animal’s tail to its nose in the chamber in order to prevent stimulation of any other parts of the body than its nose by NaCl. Furthermore, Ca^2+^ transients in ASE neurons are observed in *unc-13* and *unc-31* mutants^32^, which lack neurotransmitter and neuropeptide release, respectively, indicating that depolarization of ASE neurons was directly evoked by stimulation of ASE neurons with NaCl. Therefore, the ASE responses were solely due to stimulation of their sensory endings in the tip of the nose.

While the depolarization amplitudes of wild-type ASEL evoked by various concentrations of NaCl, which ranged from 75 mM to 150 mM, were at similar amplitudes, higher NaCl concentrations showed higher probabilities of evoking depolarization (Fig. 2A,B), indicating that membrane depolarization is an all-or-none phenomenon. Thereafter, therefore, we used a 100 mM up-step and a 50 mM down-step of NaCl concentration for stimulation of ASEL and ASER, respectively. On average, ASEL and ASER peak amplitudes induced by application of up-steps and down-steps were 38.8 ± 5.1 mV and 27.8 ± 4.3 mV, respectively (Fig. 2D). These results demonstrate that increases in NaCl concentration induce all-or-none electrical signals in ASEL.

### EGL-19, an L-type VGCC, participates in active electrical signal propagation in ASEL, but not in ASER

**Figure 3:**
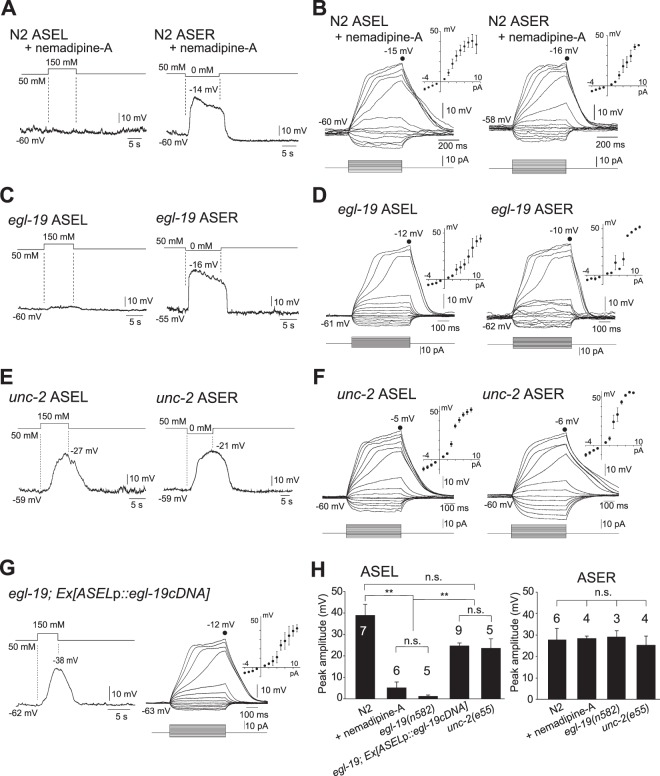
EGL-19 plays an essential role in active propagation of electrical signals in ASEL. (**A**,**B**) Membrane voltage traces of wild-type ASEL and ASER in the presence of nemadipine-A, 1.0 μM, upon stimulation with 150 mM NaCl and NaCl-free buffer, respectively, to the nose tips of animals (**A**), or upon current steps with mean *I-V* curves (ASEL: *n* = 6, ASER: *n* = 4) (**B**). (**C**,**D**) Membrane voltage traces of ASEL and ASER in *egl-19* mutants upon stimulation with 150 mM NaCl and NaCl-free buffer, respectively (**C**), or upon current injection with mean *I-V* curves (ASEL: *n* = 5, ASER: *n* = 3) (**D**). (**E**,**F**) Membrane voltage traces of ASEL and ASER in *unc-2* mutants upon stimulation with 150 mM NaCl and NaCl-free buffer, respectively (**E**), or upon current injection with mean *I-V* curves (ASEL: *n* = 5, ASER: *n* = 4) (**F**). (**G**) Membrane voltage traces of ASEL in *egl-19* transgenic animals specifically producing wild-type EGL-19 in ASEL upon stimulation with 150 mM NaCl buffer (left), or upon current injection with mean *I-V* curves (right, *n* = 9). (**H**) Mean amplitudes of membrane voltage peaks of the traces shown above in (**A**,**C**,**E**,**G**). Numbers above bars indicate numbers of animals measured. N2 data are derived from Fig. 2D. **p < 0.01, n.s., not significant by one-way ANOVA with Dunn’s test. Error bars, s.e.m.

As described above, depolarization was observed in ASEL upon an increase in environmental NaCl concentration. Bath application of nemadipine-A, an antagonist of the α1 subunit of L-type VGCCs^37^, blocked NaCl-induced depolarization of ASEL (Fig. 3A,H, left). In contrast, the response of ASER to a puff of NaCl-free buffer was not blocked by nemadipine-A (Fig. 3A,H, right). Upon current injection above a threshold level, however, supralinear depolarization was observed in both ASEL and ASER in the presence of nemadipine-A (Fig. 3B), suggesting the existence of other depolarizing voltage-gated channels than those inhibited by the drug.

**Figure 4:**
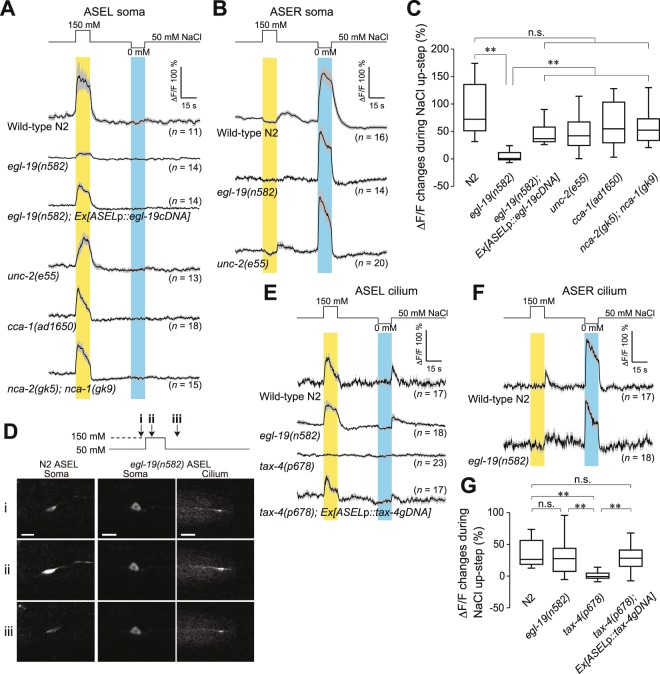
Ca^2+^ transients in cell body and sensory cilium of ASE neurons of animals immobilized in microfluidic chambers. (**A**) Ca^2+^ transients in ASEL cell bodies of wild type, *egl-19*, ASEL-specifically rescued *egl-19*, *unc-2*, *cca-1*, and a double mutant, *nca-2; nca-1*, in response to NaCl concentration changes for 15 s, which are shown on top. Grey bands, s.e.m. (**B**) Ca^2+^ transients in ASER cell bodies of wild-type, *egl-19*, and *unc-2* animals in response to NaCl concentration changes for 15 s. (**C**) Mean ΔF/F changes in ASEL cell bodies measured in (**A**) during 15-s stimulation with 150 mM NaCl buffers. Horizontal lines in boxes indicate 25th, 50th, and 75th percentiles, and whiskers represent 5th and 95th percentiles. (**D**) Upon stimulation of ASEL cilia with 150 mM NaCl buffer for 15 s, Ca^2+^ transients of ASEL cilia of *egl-19(n582)* were monitored 3 s before the stimulation (i), 5 s after the NaCl up-step (ii), and 8 s after cessation of the stimulation (iii). Note that Ca^2+^ influx into the cilia, but not to the cell body, of *egl-19* ASEL was detected. Ca^2+^ transients in the ASEL cell body of wild-type N2 are also shown as references at the left. (**E**) Ca^2+^ transients in ASEL cilia of wild type, *egl-19*, *tax-4*, and *tax-4* rescued by ASEL-specific expression of *tax-4* genomic DNA, in response to NaCl concentration changes for 15 s as shown on top. (**F**) Ca^2+^ transients in ASER cilia of wild-type and *egl-19* animals in response to NaCl concentration changes for 15 s. (**G**) Mean ΔF/F changes in ASEL cilium in (**E**) during 15-s stimulation with 150 mM NaCl buffers. Horizontal lines in boxes indicate 25th, 50th, and 75th percentiles, and whiskers represent 5th and 95th percentiles. **p < 0.01, n.s., not significant by Steel-Dwass test.

The blockade by nemadipine-A of NaCl-induced depolarization suggests that an L-type VGCC contributes to depolarization of ASEL. Since EGL-19 is the sole target of nemadipine-A among *C*. *elegans* VGCCs^37^, we examined whether ASEL depolarization in *egl-19(n582)*, a loss-of-function allele, is observed following a puff of 150 mM NaCl to its nose. As expected, NaCl-induced depolarization was not observed in ASEL of *egl-19*, like wild-type animals treated with nemadipine-A (Fig. 3C, left). In contrast, membrane depolarization was observed in *egl-19* ASER in a manner similar to that of wild type upon application of NaCl-free buffer (Fig. 3C, right). NaCl step-evoked membrane depolarizations of ASE neurons were correlated with Ca^2+^ transients induced by the same NaCl concentration steps (Fig. 4A,B). Consistent with the lack of NaCl-induced membrane depolarization in *egl-19* ASEL, Ca^2+^ imaging of cell bodies also demonstrated that *egl-19* ASEL failed to respond to stimulation with 150 mM NaCl (Fig. 4A,C), whereas *egl-19* ASER showed similar level of Ca^2+^ transients to those of wild type (Fig. 4B).

**Figure 5:**
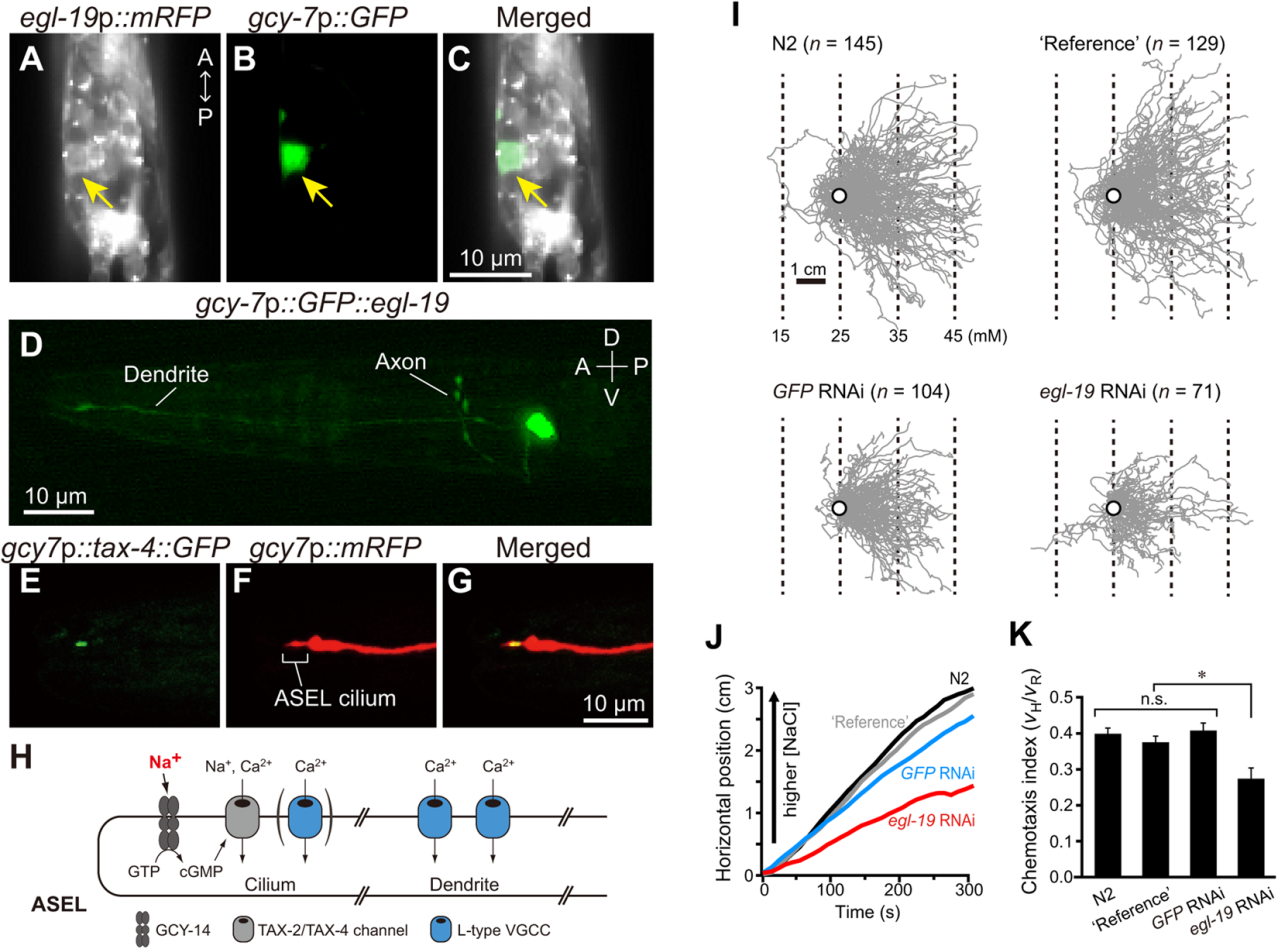
EGL-19 produced in ASEL is required for chemotaxis. (**A**–**D**) Expression patterns of *egl-19*. (**A**) mRFP production under control of the *egl-19* promoter. Note mRFP produced in ASEL as indicated by arrow. (**B**,**C**) GFP production under control of the *gcy-7* promoter. Note specific production of GFP in ASEL. (**D**) Subcellular localization of the GFP::EGL-19 fusion protein produced under control of the *gcy-7* promoter. Note specific localization of the fusion in ASEL dendrite, axon and cell soma. (**E**–**G**) Subcellular localization of the TAX-4 protein in ASEL. (**E**) TAX-4::GFP produced under control of the *gcy-7* promoter. (**F**) mRFP under control of the *gcy-7* promoter. (**G**) Merged image of (**E**,**F**). Note that TAX-4::GFP located in the chemosensory cilium, but not in dendrite, of ASEL. (**H**) Subcellular localization of membrane proteins involved in NaCl-induced signal propagation in the ASEL cilium and dendrite. (**I**) Trajectories of animals, wild-type N2, N2 producing GFP in ASEL and coelomocytes (‘Reference’), ‘Reference’ animals treated with *GFP* RNAi and ‘Reference’ animals treated with *egl-19* RNAi, on chemotaxis assay plates with an NaCl linear gradient. Open circles indicate the starting position for all trajectories of individual animals during the initial 250 s. Dotted lines indicate NaCl concentrations calculated by assuming that the gradient is linear (5 mM NaCl/cm). (**J**) Mean horizontal positions of the four strains during the NaCl chemotaxis. (**K**) Effect of knock-down of EGL-19 specifically in ASEL by RNAi on chemotactic efficiency of animals toward preferred NaCl concentrations. Chemotaxis index *v*_H_/*v*_R_, which is horizontal velocity divided by velocity along the trajectory, was computed for each strain (see Materials and methods ‘Behavioral analysis’). Each data point represents the mean ± s.e.m. One-way ANOVA with Dunn’s post-hoc test was used for statistical analysis of the data. *p < 0.05. n.s., not significant.

As described above, upon an increase in environmental NaCl concentration, EGL-19 is required for depolarization of ASEL, but not of ASER. Therefore, we examined the possibility that EGL-19 is produced in ASEL but not in ASER by observing expression of a transcriptional fusion, *egl-19*p*::mRFP*, which expresses *mRFP* under control of the *egl-19* promoter. mRFP was widely produced in most neurons, as well as body-wall and pharyngeal muscles in the head of wild-type animals (Supplementary Fig. S2A). Since mRFP co-localized with GFP produced specifically in ASEL and ASER, furthermore, both ASE neurons express *egl-19* (Fig. 5A–C and Supplementary Fig. S2B–D), indicating that asymmetry of the depolarizing activity is not due to expression of *egl-19*.

When wild-type *egl-19* cDNA was expressed specifically in ASEL of *egl-19* animals, NaCl-induced depolarization recovered in *egl-19* ASEL (Fig. 3G). Consistently, a significant increase in Ca^2+^ transients, compared to that in *egl-19*, was observed in *egl-19*, which produced wild-type EGL-19 specifically in ASEL, by stimulation with 150 mM NaCl buffer (Fig. 4A,C). Taken together, these results demonstrate that EGL-19 plays an indispensable role in electrical signal propagation in ASEL, but not in ASER, even though *egl-19* is expressed in both.

### Other channels are not required for ASEL dendritic signal propagation

Current injection induced supralinear depolarization in both ASEL and ASER of *egl-19* animals (Fig. 3D), suggesting the existence of other channels in the supralinear depolarization. In *unc-2(e55)* animals, furthermore, smaller amplitudes and slower onsets of depolarization were previously observed in RMD neurons^17^. Therefore, we also examined whether other VGCCs participate in electrical signal propagation. *unc-2* and *cca-1* genes encode α1 subunits of the R,N,P/Q- and T-type VGCCs, respectively^19,26,38,39^. ASE neurons of *unc-2* mutants showed supralinear depolarization upon depolarizing current injection in a similar manner to that of wild type (Fig. 3F), indicating that neuronal excitability of ASEL and ASER is normal in *unc-2* mutants. NaCl-induced membrane depolarization was also observed both in ASEL and ASER of *unc-2* (Fig. 3E). Upon an increase in environmental NaCl concentration, consistently, an increase in Ca^2+^ concentration was observed in the ASEL cell body of *unc-2* (Fig. 4A,C). ASEL of the *cca-1(ad1650)* mutant with a 2.5-kb genomic deletion showed a response to increased environmental NaCl concentration that was indistinguishable from that of wild type (Fig. 4A,C), indicating that T-type VGCC does not play a role in signal propagation of ASEL.

In *C*. *elegans*, *nca-1* and *nca-2* encode channels homologous to vertebrate NALCN, a voltage-independent, nonselective cation channel that acts as a Na^+^ leak channel regulating resting potentials^40,41^. In HSN neurons, NCA-1 and NCA-2 are enriched in the non-synaptic region of axons and affect synaptic transmission^42^. Therefore, we also examined whether NCA-1 and NCA-2 contribute to signal propagation in ASEL. Upon an increase in environmental NaCl concentration, normal Ca^2+^ transients were observed in ASEL cell bodies of *nca-2(gk5); nca-1(gk9)* double mutants (Fig. 4A,C), indicating that NCA-1 and NCA-2 are not essential for signal propagation in ASEL.

### Ca^2+^ influx is normal in cilia, but does not occur in cell body, of ASEL in *egl-19*

It is known that an increase in environmental NaCl concentration activates a receptor guanylyl cyclase, GCY-14, which induces Ca^2+^ influx into ASEL cilia through activation of TAX-2/TAX-4 cGMP-gated channels^30^ by increasing cGMP concentration. This Ca^2+^ influx is likely to induce depolarization of the cilium membrane potential, since NaCl application to the nose tip of *tax-4* mutants failed to evoke membrane depolarization of ASEL (Supplementary Fig. S3B,C). This is consistent with specific subcellular localization of TAX-4 to ASEL cilia (Fig. 5E–G). The cilium membrane depolarization may in turn activate VGCCs to propagate the electrical signal from the cilium to the cell body of ASEL, since NaCl application to the nose tip of *egl-19* mutants failed to evoke membrane depolarization of ASEL (Fig. 3C, left). Consistently, the NaCl application also failed to cause Ca^2+^ influx into the cell body of *egl-19* mutants (Fig. 4A,C).

To test the scenario that EGL-19 plays an essential role in active propagation of dendritic electric signals in ASEL, we measured Ca^2+^ transients of ASEL and ASER cilia upon increases and decreases in environmental NaCl concentration. Ca^2+^ transients were clearly observed in ASEL and ASER cilia of both wild type and *egl-19* upon an increase and a decrease in environmental NaCl concentration, respectively (Fig. 4D–G), demonstrating that Ca^2+^ influx into cilia occurs in *egl-19* mutants.

In contrast, no Ca^2+^ influx into ASEL cilia nor depolarization of ASEL was observed in *tax-4* mutants (Fig. 4E,G and Supplementary Fig. S3). ASEL-specific expression of the wild-type *tax-4* gene rescued the defect of Ca^2+^ influx into the cilia in *tax-4* (Fig. 4E,G), consistent with mediation of Ca^2+^ influx by TAX-2/TAX-4 cGMP-gated channels.

Unlike wild type, however, an increase in Ca^2+^ concentration in the cell body could not be observed in *egl-19* (Fig. 4A,C,D), suggesting that Ca^2+^ influx through TAX-2/TAX-4 channels induces depolarization of the ASEL cilium membrane, which in turn activates the EGL-19 VGCC for ASEL dendritic electrical signal propagation from the cilia to the cell body. Consistent with this finding, TAX-4 was specifically localized to the ASEL cilium, and EGL-19 was found located in the dendrites, axons and cell body of ASEL (Fig. 5D–H). Since supralinear depolarization of *egl-19* ASEL was observed upon depolarizing current injection, VGCCs other than EGL-19 are likely to function in ASEL.

### EGL-19 in ASEL is required for chemotaxis along NaCl gradients

The results described above suggest that EGL-19 plays a role in *C*. *elegans* chemotaxis along NaCl gradients. ASEL activation increases the probability of forward locomotion upon an increase in environmental NaCl concentration, while ASER activation increases probability of backward locomotion followed by a pirouette, a large-angle turn, upon a decrease in environmental NaCl concentration^32,43^.

As described above, an increase in environmental NaCl concentration failed to activate ASEL of *egl-19* mutants due to the lack of dendritic electrical signal propagation. Therefore, *egl-19* mutants are likely to be inefficient in chemotaxis toward preferred NaCl concentrations, although functional ASER less efficiently brings animals to preferred NaCl concentrations than wild type. To test this possibility, we analyzed chemotactic speeds and chemotaxis indices of wild-type animals in which EGL-19 was ASEL-specifically knocked down using RNA interference (RNAi) (Fig. 5I–K).

The ‘run’ speeds of animals treated with ASEL-specific RNAi against *egl-19* or *GFP* were statistically significantly slower than those of wild type and ‘reference’ animals untreated with RNAi (Supplementary Fig. S4A; see Materials and Methods for the definition of ‘run’ and ‘reference’). This slow locomotion may be due to the small body size of animals treated with ASEL-specific RNAi (Supplementary Fig. S4B), because ASE neurons release hormones required for normal body size^44,45^. The chemotaxis index of animals with EGL-19 knocked down by ASEL-specific RNAi was statistically significantly lower than that of wild-type and ‘reference’ animals, including one in which GFP was knocked down with ASEL-specific RNAi (Fig. 5K). These results are consistent with vital roles for both ASEL and ASER in chemotaxis of animals along NaCl gradients, and non-functionality of ASEL due to the lack of dendritic electrical signal propagation significantly affects chemotactic behavior.

## Discussion

### Active propagation of dendritic electrical signals in ASEL

The ASE neuron pairs, ASEL and ASER, are required for *C*. *elegans* chemotaxis toward preferred concentrations of NaCl, and are activated by increases and decreases in NaCl concentration, respectively. In the present study, we analyzed activity of ASE neurons using *in vivo* whole-cell patch-clamp recordings and Ca^2+^ imaging of their somata and cilia. We found that: (1) In response to depolarizing current steps, supralinear depolarization of both ASEL and ASER occurred (Fig. 1C). This non-linear depolarization was blocked when both Na^+^ and Ca^2+^ ions were removed from the bath solution (Fig. 1F). (2) Upon stimulation of an animal’s nose with various concentrations of NaCl, all-or-none membrane depolarization was observed in ASEL (Fig. 2A,B). (3) The EGL-19 VGCC plays an essential role in the all-or-none depolarization of ASEL, but not in ASER (Fig. 3A,C,G,H). (4) Upon an increase in environmental NaCl concentration, Ca^2+^ influx into the ASEL cilium through the TAX-2/TAX-4 cGMP-dependent channel is normal in *egl-19* mutants (Fig. 4D,E,G). (5) TAX-4 exists only in the cilia of ASEL, and EGL-19 exists in the dendrite, cell body and axon of ASEL (Fig. 5D–G). (6) Wild-type animals in which EGL-19 was cell-specifically knocked down in ASEL with RNAi were partially deficient in chemotaxis toward preferred concentrations of NaCl (Fig. 5I–K).

These results are consistent with the following scenario (Fig. 5H): The receptor guanylyl cyclase GCY-14 of ASEL cilia is one of the sensor molecules that detects an increase in environmental Na^+^ ion concentrations and produces cGMP^30,31^. Upon activation of the cGMP-gated TAX-2/TAX-4 channel through an increase in cGMP concentration produced by GCY-14, Ca^2+^ influx occurs in ASEL cilia, resulting in ciliary membrane depolarization. Consistently, membrane depolarization was not observed in *tax-4* mutants when the nose tip was stimulated with NaCl (Supplementary Fig. S3B,C). The cilium membrane depolarization is likely to open EGL-19 VGCCs in ASEL dendrites, since NaCl-induced membrane depolarization was not observed in ASEL of wild-type animals treated with nemadipine-A, nor in *egl-19* mutants (Fig. 3A,C). Thus, electric signals actively propagate to the ASEL cell body from its cilium through its dendrite.

When stimulated with NaCl-free buffer, in contrast, membrane depolarization of ASER was clearly observed in wild-type animals treated with nemadipine-A and in *egl-19* mutants (Fig. 3A,C, right), suggesting that the EGL-19 VGCC is not required for dendritic signal propagation in ASER. This is consistent with the previous observation that ASER is isopotential and depolarization can spread passively^16^. Upon depolarizing current injection, supralinear depolarization was observed in ASEL and ASER of wild-type animals treated with nemadipine-A, *egl-19* and *unc-2* mutants (Fig. 3B,D,F), suggesting that EGL-19, UNC-2, and other VGCCs act redundantly in electric signal propagation in ASEL and ASER.

The lack of active propagation of dendritic electrical signals in ASEL negatively affected chemotaxis toward preferred concentrations of NaCl, and animals were less effectively attracted to preferred concentrations than were wild-type animals (Fig. 5I–K). As described above, increased environmental NaCl concentration activates ASEL for forward locomotion, while decreased NaCl concentration activates ASER for reverse locomotion. While animals can locate preferred concentrations of NaCl with only one of these two sensory neurons, activation of both neurons enables animals to find preferred concentrations more effectively.

### All-or-none regenerative propagation of sensory signals in dendrites

ASEL detects various water-soluble ions in the environment, including Na^+^, Li^+^, Mg^2+^ and alkaline pH^30,31,45^, and ASER senses nearly all salts including K^+^, Br^−^, I^−^ and the amino acid, methionine^31,46,47^. A receptor guanylyl cyclase, GCY-14, of ASEL cilia senses both Na^+^ and alkaline pH^30^. Therefore, multiple chemicals are sensed by a single sensor molecule, and many chemicals are detected by a single sensory neuron in *C*. *elegans*. How does *C*. *elegans* respond to only significant environmental changes mixed with sensory noise from other ions? By setting the threshold that evokes regenerative depolarization in dendrites of sensory neurons, *C*. *elegans* may be able to respond to only significant environmental changes among many others.

While a 100-mM increase in NaCl concentration reliably evoked membrane depolarization in ASEL, NaCl concentration changes ranging from 75 mM to 25 mM evoked depolarization less consistently (Fig. 2A,B). However, *C*. *elegans* could detect shallower NaCl concentration gradients during chemotaxis (Fig. 5I). Sensitivity of the GCY-14 receptor guanylyl cyclase to NaCl may change during chemotaxis along NaCl gradients. Guanylyl cyclase activity is regulated by upstream kinase homology domains (KHD), the stability of which is modified by phosphorylation^45,48–50^. Therefore, GCY-14 sensitivity to NaCl may be dependent upon the phosphorylation states of the receptor KHD. Furthermore, sensory activity of ASEL is also regulated by downstream effector proteins of GCY-14^30^, which include EGL-4, a cGMP-dependent protein kinase (PKG)^51^, phosphodiesterases^52,53^, TAX-6, a homolog of calcineurin A, and NCS-1, a neuronal calcium sensor^54–56^. These downstream effectors may also modulate ASEL activity during the chemotaxis.

### *C*. *elegans* neurons send electrical signals both actively and passively

As described above, both ASEL and ASER were supralinearly depolarized in response to depolarizing current steps. This non-linear depolarization was blocked when both Na^+^ and Ca^2+^ ions were removed from the bath solution. By injecting current pulses, similarly, RMD neurons were nonlinearly depolarized, and showed bistability following cessation of the current step^17^. Furthermore, olfactory AWA neurons seem to actively send electrical signals, since an increase in Ca^2+^ concentration in the AWA cell body upon diacetyl application is abolished by nemadipine-A treatment and *egl-19* mutation^57,58^.

Upon current injection, supralinear depolarization was observed in both ASEL and RMD of *egl-19*, which encodes an L-type VGCC, and *unc-2*, which encodes an R,N,P/Q-type VGCC. Furthermore, normal increases in Ca^2+^ concentration were also observed in *cca-1*, which encodes a T-type VGCC, or in *nca-2; nca-1* double mutants, both of which encode channels homologous to vertebrate NALCN, a voltage-insensitive, non-selective cation channel^40,41^. These results suggest that multiple classes of Ca^2+^ channels, which include yet unidentified channels, contribute redundantly to the supralinear depolarization.

It is now apparent that upon stimulation with NaCl, the *C*. *elegans* nervous system actively propagates electrical signals. In addition to ASER, motor neurons and AVA interneurons propagate electrical signals passively^9,17^. As in mammalian brain, therefore, both active and passive propagation of electric signals occur in the *C*. *elegans* nervous system.

## Materials and Methods

### Strains

*C*. *elegans* strains used in this study were obtained from the *Caenorhabditis* Genetics Center (University of Minnesota, Minneapolis, MN). Animals were grown to adulthood on nematode growth medium (NGM) (50 mM NaCl, 2% agar, 2.5% peptone, 5 mg/L cholesterol, 1.0 mM CaCl_2_, 1.0 mM MgSO_4_, 25 mM potassium phosphate, pH 6.0) seeded with *Escherichia coli* OP50 at 20 °C using standard methods^59^. Strains used in this work are listed in Supplementary Table S1.

### Electrophysiology

After hatching, Day-4 hermaphrodite animals were immobilized on a circular (12 mm in diameter) cover glass, which was coated with Sylgard 184 (Dow Corning, Midland, MI), with a glue (Histoacryl Blue, B. Braun, Melsungen, Germany). The cover glass was transferred to a recording chamber placed on the stage of an upright microscope with a 60x water-immersion objective (model BX50WI; Olympus), and was perfused (2–3 mL/min) with ECS (see below). A small piece of a cuticle and body wall of an animal’s head was cut out with a sharp glass needle, and ASE cell bodies producing GFP were brought out from the head. Borosilicate glass pipettes (GC150TF-10; Harvard Apparatus, UK) with a tip resistance of 6–8 MΩ were used as electrodes for current-clamp recordings in a whole-cell patch- clamp configuration.

Patch pipettes were pulled on a DMZ universal puller (Zeitz Instruments, Munich, Germany). Recordings were performed with a Multiclamp 700B amplifier (Molecular Devices, Foster City, CA) and the Clampex software (Molecular Devices). Recordings were corrected for junction potentials, and series resistances were compensated by 50%. Recordings with 1–15 GΩ seal resistances were normally observed, and recordings with seal resistances less than 1.0 GΩ were discarded. Before the compensation, recordings with 20–70 MΩ series resistances were normally observed, and recordings with higher than 100 MΩ were discarded. Data were acquired at 15 kHz and filtered at 2.4 kHz.

NaCl stimulation of living animals was carried out by puffing a designated concentration of NaCl from a pipette (tip pore size: <10 µm), using Pico Pump (model PV830; World Precision Instruments, Sarasota, FL). The puff capillary tip was placed within the range of 50 µm from animal’s nose, and NaCl buffer was puffed for at least 5 s as shown in Fig. 1B.

The following solutions were used for electrophysiological recordings: ECS consisted of 50 mM NaCl, 150 mM sucrose, 10 mM glucose, 15 mM HEPES, 5 mM CaCl_2_, 1.0 mM MgCl_2_, and 5 mM KCl (pH 7.2, ~330 mOsm). For Na^+^- or Ca^2+^-removed ECS, 50 mM NaCl or 5 mM CaCl_2_ were removed, respectively, from normal ECS. For Na^+^- or Ca^2+^-replaced ECS, 50 mM NaCl or 5 mM CaCl_2_ was replaced with 50 mM NMDG or 5 mM MgCl_2_, respectively. For Na^+^/Ca^2+^-removed ECS, 50 mM NaCl and 5 mM CaCl_2_ were removed from normal ECS. For Na^+^/Ca^2+^ replaced-solution, both of 50 mM NaCl and 5 mM CaCl_2_ were replaced with 50 mM NMDG and 5 mM MgCl_2_. To Ca^2+^-removed or Ca^2+^-replaced ECS, 5 mM EGTA was added. For NaCl stimulation, ECS containing 0 mM, 75 mM, 100 mM, 125 mM, or 150 mM NaCl was puffed to the animal’s nose. To apply nemadipine-A to animals, ECS containing 1.0 μM nemadipine-A was perfused. Osmolarity of all the ECS described above was adjusted to ~330 mOsm with sucrose dissolved in doubly deionized H_2_O.

The recording pipette solution contained 115 mM K-gluconate, 15 mM KCl, 5 mM MgCl_2_, 0.25 mM CaCl_2_, 10 mM HEPES, 20 mM sucrose, 5 mM EGTA, 5 mM Mg_2_ATP, 0.5 mM Na_2_GTP, and was adjusted to pH 7.2 and 315 mOsm. To visually observe neuronal cell damage, 10 μM sulforhodamine was included in pipette solution. Chemical reagents were obtained from either Sigma Aldrich (St. Louis, MO) or Wako Chemical (Osaka, Japan).

### Chemotaxis assay

One day before assay, Day-4 animals (4 days after hatching) were selected and transferred to new NGM plates seeded with OP50. The following animals were used for chemotaxis assay: Wild-type N2; OF1201 (‘Reference’), which cell-specifically produces GFP in ASEL and coelomocytes; OF1202 (‘*GFP* RNAi’), which cell-specifically produces GFP in coelomocytes, but not in ASEL, and mRFP in the tail; and OF1203 (‘*egl-19* RNAi’), which cell-specifically produces GFP in coelomocytes, but not in ASEL, and mRFP in the tail.

Immediately before assay, 5–10 animals were transferred to a chemotaxis (CTX) agar plate, containing 1.5% agar, 1.0 mM CaCl_2_, 1.0 mM MgSO_4_, 5 mM potassium phosphate, pH 6.0, with a worm picker, and allowed to run freely on the agar surface in order to remove attached bacteria. After ~30 s, animals were transferred to the center of an NaCl gradient plate using a worm picker. Movements of the animals were recorded at 5 frames/s with a CCD camera (INFINITY3-6URM; Lumenera, Canada) for 10 min. The chemotaxis assay was performed at 20 °C.

NaCl gradient plates were made in square dishes (10 cm × 10 cm; Thermo Fisher Scientific) in a slanted position (with one side ~5-mm higher than the other) by pouring 25 mL of melted CTX agar containing 50 mM NaCl, 1.5% agar, 1.0 mM CaCl_2_, 1.0 mM MgSO_4_, 5 mM potassium phosphate, pH 6.0 in the plate. After solidifying, the dish was laid flat and filled with 25 mL melted CTX agar containing 1.5% agar, 1.0 mM CaCl_2_, 1.0 mM MgSO_4_, 5 mM potassium phosphate, pH 6.0. The dish was kept open without a lid for 1.0 hr at room temperature for drying. The plate was used for chemotaxis assay 16–22 hrs after casting.

### Behavioral analysis

Animal trajectories on CTX agar plates were captured with an ImageJ plug-in (MosaicSuite; MOSAIC Group, Dresden, Germany), and analyzed using a custom R script (https://www.r-project.org/). To quantify chemotactic performance of each strain, we calculated a non-dimensional index for every trajectory: the mean horizontal velocity of animals along the NaCl gradient, <*v*_H_>, divided by the mean ‘run’ speed along the trajectory, <*v*_R_>, as described previously^60^. We defined ‘run’ as continuous locomotion for more than 3 s without interruption by a turn in which a directional change larger than 60° occurs for over 1.2 s. Trajectories between 150 s and 250 s were used for the calculation of chemotaxis index since ‘run’ speeds became relatively constant at 150 s after placing animals on CTX plates (Supplementary Fig. S5). At 250 s, animals started to reach the wall of the plate.

### Statistical analysis

Statistical analysis was performed using Sigma Plot v13 (Systat Software Inc.) or R v3.4.3. For data analysis of electrophysiology and behavioral assays, one-way ANOVA with Dunn’s post-hoc test was used. For analysis of Ca^2+^ imaging data, the Steel-Dwass test was used for multiple comparisons. All data points are means ± s.e.m., unless otherwise stated.

### Note added

During revision of the manuscript, all-or-none action potentials evoked by current injection was observed in the AWA olfactory neuron^61^.

## Supplementary information

Supplementary Information

## Data Availability

All data generated or analyzed during this study are included in this published article and its Supplementary Information files.
