# Supplementary material for: Active propagation of dendritic electrical signals in C. elegans
Source: Sci Rep. 2019 Mar 5;9:3430. doi: 10.1038/s41598-019-40158-9 (PMC6401061; doi:10.1038/s41598-019-40158-9)
Supplement: Supplementary file 1 — Supplementary Information [file 41598_2019_40158_MOESM1_ESM.docx]

**Supplementary Information**

**Active propagation of dendritic electrical signals in *C. elegans***

**Tomomi Shindou^1*^, Mayumi Ochi-Shindou^1*^, Takashi Murayama^2*^, Ei-ichiro Saita^2^, Yuto Momohara^2^, Jeffery R. Wickens^1^ & Ichiro N. Maruyama^2^**

^1^Neurobiology Research Unit, ^2^Information Processing Biology Unit,

Okinawa Institute of Science and Technology Graduate University, Okinawa 904-0495, Japan

**Supplementary Materials and Methods**

**PCR products and germline transformation.** Oligonucleotide primers used in this work are listed in Supplementary Table S2. A 9.8-kb genomic DNA fragment, which contains 7.9-kb sequences upstream of the *egl-19b* gene encoding the longest isoform, was amplified with Primers A and B. A DNA fragment encoding *mRFP::unc-54 3’-UTR* was amplified from pHK-mRFP (a gift from Hiroshi Kagoshima, National Institute of Genetics, Mishima, Japan) with Primers C and D, in which the *egl-19* promoter sequence is underlined. PCR was performed using high-fidelity Taq polymerase, PrimeSTAR HS or Tks Gflex (Takara Bio, Shiga, Japan). The resulting two DNA fragments were joined by overlap extension PCR (OE-PCR)^1^ with Primers A and D, resulting in *egl-19*p*::mRFP*.

A 5.6-kb *egl-19b* cDNA was amplified by PCR with Primers E and F, using as a template, a cDNA library prepared from mixed culture of wild-type N2. Two DNA fragments, *gcy-7*p*::GFP* and *unc-54* 3’-UTR, were amplified from p*gcy-7*p::*GFP* plasmid DNA^2^ by PCR with Primers G and H, and Primers I and D, respectively. These resulting two PCR products have common sequences to the *egl-19* cDNA as shown by underlined sequences of Primers H and I. From these three PCR products, OE-PCR with Primers G and D produced *gcy-7*p*::GFP::egl-19 cDNA::unc-54 3’-UTR*. To make *gcy-7*p*::egl-19 cDNA*, the 5.6-kb *egl-19b* cDNA PCR product was cloned by SLIC assembly^3^ into a vector containing *gcy-7* promoter and *unc-54* 3’-UTR, which were amplified from p*gcy-7*p*::GCaMP3*^4^ with Primers I and J. Underlined sequences of these Primers overlap with the *egl-19* cDNA sequence.

DNA fragments for ASEL-specific *egl-19* RNAi were prepared as previously described^5^. A 1.2-kb *gcy-7* promoter region was amplified by PCR with Primers K and L, using p*gcy-7*p::*GFP* plasmid DNA as a template. A 2-kb DNA fragment encoding part of *egl-19* cDNA was amplified with primer pairs, Primers M and N, and Primers O and P, to make sense and antisense *egl-19* RNAi constructs, designated *egl-19 RNAi(sense)* and *egl-19* *RNAi(antisense)*. The resulting PCR products were joined by OE-PCR to make *gcy-7*p*::egl-19 RNAi(sense)* and *gcy-7*p*::egl-19 RNAi(antisense)*.

ASEL-specific *GFP* RNAi constructs, *gcy-7*p*::GFP RNAi(sense)* and *gcy-7*p*::GFP RNAi(antisense)*, were also made as above with primer pairs, Primers Q and R, and Primers S and T. These sense and antisense constructs overlap with the 864-bp *GFP* sequence encoded by the pPD95.77 vector. A PCR fragment of *gcy-7*p*::tax-4gDNA* was amplified from ASEL-specific *tax-4* expression plasmid^4^ with primers U and V, and was cloned into pPD95.79 between *Sph* I and *Sma* I restriction sites, resulting in *gcy-7*p*::tax-4gDNA::gfp*.

Transgenic animals were generated by germline transformation as described previously^6^. A plasmid or PCR DNA was injected at concentrations of 0.3-50 ng/μL, together with 20-50 ng/μL of a marker plasmid, p*lin-44*p*::mRFP*, p*gcy-7*p*::GFP* or p*unc-122*p*::GFP*. To identify ASE neurons, N2 carrying *ixEx225[egl-19*p*::mRFP]* was mated with OH3191 and OH3192 to make *otIs3;* *ixEx225* and *ntIs1; ixEx225* animals, respectively. For ASEL-specific *egl-19* RNAi, 20 ng/μL each of the sense and antisense constructs was co-injected with p*lin-44*p*::mRFP* into N2 carrying *ixEx232[gcy-7*p*::GFP; unc-122*p*::GFP]*.

**Ca^2+^ imaging.** Ca^2+^ imaging in ASEL and ASER was performed as described^4^ with modifications. GCaMP3-expression constructs, p*gcy-7*p*::GCaMP3* and p*gcy-5*p*::GCaMP3*, were used for germline transformation in order to make animals cell-specifically produce GCaMP3 in ASEL and ASER, respectively. Animals were loaded into a custom-designed microfluidic device of polydimethylsiloxane with loading buffer (5 mM potassium phosphate, pH 6.0, 1.0 mM MgSO_4_, 1.0 mM CaCl_2_, and 50 mM NaCl, adjusted to 350 mOsm with sorbitol), and then were stimulated with loading buffer that contained 150 mM NaCl (150 mM NaCl buffer) or no NaCl (NaCl-free buffer) so as to cause a 100-mM increase or a 50-mM decrease of environmental NaCl concentrations, respectively.

To reduce head movement during imaging of cilia, 0.1% 1-phenoxy-2-propanol (1p2p) was added to loading buffer, and imaging was started 10-30 min after an animal was immobilized in the microfluidic chamber. In the presence of 0.1% 1p2p, wild-type ASE neurons responded NaCl concentration changes indistinguishably from those in the absence of the anesthesia.

A series of TIFF images were captured at 2 frames/s using a Zeiss LSM 510 confocal microscope with a Plan-Apochromat water-immersion objective (40x, NA 1.2). In a series of fluorescent images taken from each animal, the target cell position of each frame was determined, and fluorescence intensity of a region of interest (ROI) was analyzed using Matlab software. The ROI was defined by highlighting desired cells, and the same number of pixels near the target cell was used to measure the background fluorescence of the animal. In each image, mean fluorescence intensity of the background ROI was subtracted from that of the target cell. Mean fluorescence intensity of a series of 10 frames immediately before an NaCl concentration shift was set as F, and a relative fluorescence intensity increase in percentage was calculated as ΔF/F.

**References**

1. Higuchi, R., Krummel, B. & Saiki, R. A general method of *in vitro* preparation and specific mutagenesis of DNA fragments: study of protein and DNA interactions. *Nuc. Acids Res.* **16**, 7351-7367 (1988).

2. Yu, S., Avery, L., Baude, E. & Garbers, D.L. Guanylyl cyclase expression in specific sensory neurons: a new family of chemosensory receptors. *Proc. Natl. Acad. Sci. U S A* **94**, 3384-3387 (1997).

3. Li, M.Z. & Elledge, S.J. Harnessing homologous recombination *in vitro* to generate recombinant DNA via SLIC. *Nat. Methods* **4**, 251-256 (2007).

4. Murayama, T., Takayama, J., Fujiwara, M. & Maruyama, I.N. Environmental alkalinity sensing mediated by the transmembrane guanylyl cyclase GCY-14 in *C. elegans*. *Curr. Biol.* **23**, 1007-1012 (2013).

5. Esposito, G., Di Schiavi, E., Bergamasco, C. & Bazzicalupo, P. Efficient and cell specific knock-down of gene function in targeted *C. elegans neurons*. *Gene* **395**, 170-176 (2007).

6. Mello, C.C., Kramer, J.M., Stinchcomb, D. & Ambros, V. Efficient gene transfer in *C. elegans*: extrachromosomal maintenance and integration of transforming sequences. *EMBO J.* **10**, 3959-3970 (1991).

**Supplementary Table S1.** *C. elegans* strains used

Wild-type Bristol N2 strain

MT1212 *egl-19(n582) IV*

CB55 *unc-2(e55) X*

OH3191 *otIs3[gcy-7prom::gfp; lin-15(+)]*

OH3192 *ntIs1[gcy-5prom::gfp; lin-15(+)]*

OF829 *ixEx144[gcy-7*p*::GCaMP3; lin-44*p*::mRFP]*

OF830 *ixEx145[gcy-5*p*::GCaMP3; lin-44*p*::mRFP]*

OF1079 *unc-2(e55); otIs3[gcy-7prom::gfp; lin-15(+)]*

OF1080 *unc-2*(*e55*)*; ntIs1[gcy-5prom::gfp; lin-15(+)]*

OF1081 *egl-19(n582); otIs3[gcy-7prom::gfp; lin-15(+)]*

OF1082 *egl-19(n582); ntIs1[gcy-5prom::gfp; lin-15(+)]*

OF1085 *unc-2(e55); ixEx221[gcy-7*p*::GCaMP3; lin-44*p*::mRFP]*

OF1086 *unc-2(e55); ixEx222[gcy-5*p*::GCaMP3; lin-44*p*::mRFP]*

OF1087 *egl-19(n582); ixEx223[gcy-7*p*::GCaMP3; lin-44*p*::mRFP]*

OF1088 *egl-19(n582); ixEx224[gcy-5*p*::GCaMP3; lin-44*p*::mRFP]*

OF1130 *ixEx225[egl-19*p*::mRFP]*

OF1131 *otIs3; ixEx225*

OF1132 *ntIs1; ixEx225*

OF1133 *ixEx226[gcy-7*p*::GFP::egl-19 cDNA; lin-44*p*::mRFP]*

OF1173 *cca-1(ad1650); ixEx228[gcy-7*p*::GCaMP3; lin-44*p*::mRFP]*

OF1174 *nca-2(gk5); nca-1(gk9); ixEx229[gcy-7*p*::GCaMP3; lin-44*p*::mRFP]*

OF1175 *egl-19(n582); ixEx230[gcy-7*p*::egl-19 cDNA; gcy-7*p*::GCaMP3;* *lin-*

*44*p*::mRFP]*

OF1200 *egl-19(n582); ixEx231[gcy-7*p*::egl-19 cDNA; gcy-7*p*::gfp]*

OF1201 *ixEx232[gcy-7*p*::gfp; unc-122p::gfp]*

OF1202 *ixEx232[gcy-7*p*::gfp; unc-122*p*::gfp]; ixEx233[gcy-7*p*::gfp(RNAi);*

*lin-44*p*::mRFP]*

OF1203 *ixEx232[gcy-7*p*::gfp; unc-122*p*::gfp]; ixEx234[gcy-7*p*::egl-19(RNAi);*

*gcy-7*p*::gfp(RNAi); lin-44*p*::mRFP].*

OF1259 *tax-4(p678); otIs3[gcy-7prom::gfp; lin-15(+)]*

OF1293 *ixEx255[gcy-7*p*::tax-4gDNA::gfp; gcy-7*p*::mRFP; lin-44*p*::mRFP]*

**Supplementary Table S2.** Oligonucleotide primers used

Primer A, 5’-gttgcacagatttgtctgcagccgccaccttc

Primer B, 5’-tggtggatggattcaaaaagtggatccctttg

Primer C, 5’-ctttttctctctcaataagatccaaagggatccccgggattgg

Primer D, 5’-gggcccgtacggccgactagtaggaaacag

Primer E, 5’-atgtcagtgttagcgagtatgatgtc

Primer F, 5’-tcaaagagttgtaactaaaagtagatcttcttgtga

Primer G, 5’-ggaaacagctatgaccatgattacgccaag

Primer H, 5’-gatgacatcatactcgctaacactgacattttgtatagttcatccatgccatg

Primer I, 5’-ttttagttacaactctttgagatatctgagctccgcat

Primer J, 5’-catactcgctaacactgacatgctagccaagggtcctcctg

Primer K, 5’-ttgcatgctacagttctttctaacat

Primer L, 5’-ggatcccccgggattattttcttatgctaaac

Primer M, 5’-taatcccgggggatcctcagtgttagcgagtatgatg

Primer N, 5’-cagctggctagcgattcaattccatgatacattac

Primer O, 5’-tctagagaattctcagtgttagcgagtatgatg

Primer P, 5’-taatcccgggggatccgattcaattccatgat

Primer Q, 5’-taatcccgggggatccagtaaaggagaagaacttttcac

Primer R, 5’-tttgtatagttcatccat

Primer S, 5’-gctagccagctgagtaaaggagaagaacttttcac

Primer T, 5’-taatcccgggggatcctttgtatagttcatccat

Primer U, 5’-caacttggaaatgaaataagcttgcatgctacagttctttctaacatgtaatatttg

Primer V, 5’-ctttgggtcctttggccaatcccgggtttgagcaaggattcagattcagttc

Underlines indicate overlap sequences for cloning or OE-PCR.

**Supplementary Figure S1.** Membrane voltage changes in response to current steps of ASEL in wild-type N2. Current-clamp recordings of an animal were carried out three times consecutively, the first and third in normal ECS (top and bottom), and the second in ECS, which Na^+^ was replaced with NMDG^+^ (Na^+^-replaced ECS) (**A**), and in which Ca^2+^ was replaced with Mg^2+^ (Ca^2+^-replaced ECS) (**B**). These are representatives of three independent recordings.

**Supplementary Figure S2.** Expression patterns of the *egl-19* gene. (**A**) Expression of *egl-19*p*::mRFP* in the wild-type head region. Note that in wild-type animals, mRFP was produced in neurons, body-wall muscles and pharynx. (**B**-**D**) Expression of the *egl-19* gene in ASER. (**B**) Expression of *mRFP* under control of the *egl-19* promoter. (**C**) *GFP* expression under control of the ASER-specific *gcy-5* promoter.

**Supplementary Figure S3.** Neural responses of *tax-4* ASEL. (**A**) Membrane voltage changes in response to current steps and average *I-V* curve. A representative of six independent measurements. (**B**) A representative of six independent membrane voltage traces upon an increase in environmental NaCl concentration. (**C**) Mean amplitudes of membrane voltage peak upon NaCl stimulation shown in (B). N2 data were derived from Fig. 2D in the main text. ***p* <0.01 by Mann-Whitney U test (*n* = 6). Error bars, s.e.m.

**Supplementary Figure S4.** ‘Run’ speed and body length of *C. elegans* during chemotaxis along NaCl gradients. (**A**) ‘Run’ speed (v_R_) of strains on NaCl chemotaxis plates, which were measured from chemotaxis assay movies. Horizontal lines of each box indicate 25th, 50th, and 75th percentiles, and whiskers represent 5th and 95th percentiles. One-way ANOVA with Dunn’s post-hoc test was used to compare the data. (**B**) Body lengths of strains on NaCl chemotaxis plates. Body lengths of the four strains were measured from animal images in the NaCl chemotaxis assay movies. The length of longitudinal center line of each animal was measured three times using an ImageJ function, and were averaged to obtain mean body lengths of the four strains. All animals in the movies were analyzed. Horizontal lines of each box indicate 25th, 50th, and 75th percentiles, and whiskers represent 5th and 95th percentiles. Body lengths of the strains (mean ± s.d.) were 1.22 ± 0.06 (N2, *n* = 26), 1.24 ± 0.10 (‘Reference’, *n* = 23), 1.11 ± 0.07 (*GFP* RNAi, *n* = 22), and 1.14 ± 0.05 (*egl-19* RNAi, *n* = 22). One-way ANOVA with Dunn’s post-hoc test was used for statistical analysis of the data. ***p* < 0.005; n.s., not significant.

**Supplementary Figure S5.** Mean ‘run’ speeds of four strains during chemotaxis along NaCl gradients as described in Materials and Methods in the main text. Mean ‘run’ speeds were calculated every 10 s.
